# Employees’ opinions on critical success factors and performance criteria in disaster and emergency management

**DOI:** 10.12669/pjms.35.6.778

**Published:** 2019

**Authors:** Saime Sahinoz, Unal Yaprak, Turgut Sahinoz

**Affiliations:** 1 Prof. Dr. Saime Sahinoz, MD. Public Health Specialist, Department of Disaster Management, Faculty of Health Sciences, Gumushane University, Gumushane, Turkey; 2 Unal Yaprak, PhD Student, Department of Disaster Management, Faculty of Health Sciences, Gumushane University, Gumushane, Turkey; 3 Dr. Turgut Sahinoz, MD. Assistant Professor, Department of Health Management, Public Health Specialist, Faculty of Health Sciences, Ordu University, Ordu, Turkey

**Keywords:** Critical success factors, Disaster, Emergency, Performance

## Abstract

**Objective:**

To evaluate the critical success factors at disaster and emergency management and to analyse their impact on performance to guide the managers and decision makers.

**Methods:**

This was a cross-sectional study. It included all staff working in the fire brigade, 112 Emergency, Disaster and Emergency Management Authority and Red Crescent institutions serving in emergency situations in five provinces (Rize, Artvin, Trabzon, Gümüşhane, Bayburt) in the Eastern Black Sea region of Turkey. One province (Gümüşhane) was selected by cluster sampling method. Questionnaire which is the data collection method in the study was applied between 08/01/2016 - 02/02/2016 to all of the staff working in the fire brigade, 112 Emergency, Disaster and Emergency Management Authority and Red Crescent institutions serving in emergency situations in Gümüşhane province.

**Results:**

It has been found out that there is a significant and positive relationship between all of the critical success factors, planning, response and recovery criteria, and performance variance.

**Conclusions:**

The presence of well-planned emergency aid systems in the institutions or organizations involved in this area is an element of success. For this reason, this factor should be given more importance.

## INTRODUCTION

Emergency management is sometimes used interchangeably with the term disaster management, especially in the context of biological and technological hazards and health emergencies. However, although there is a high degree of overlap, an emergency is defined as a dangerous event that does not seriously interfere with the functioning of a community or community.^1^ Disaster management is; to be able to master extraordinary or unusual situations, so that they do not have a disaster Dynamics.^2^

Millions of people are affected every year in the world and in our country due to disasters.Many casualties and injuries occur, and billions of dollars of economic losses are occurring due to disasters. The population under the world’s disaster risk increases by 70-80 million every year.^2^

In addition, 70% of our country lands are on the first and second earthquake zone. From 1950 to 2014 about 34 thousand people lost their lives due to earthquakes.^3^ According to The International Disaster Database (EM-DAT), over the period 1986-2016, 1675825 people have lost their lives due to natural and technological disasters worldwide, a total of about four billion people have been affected and billions of dollars of economic losses have been experienced.^4^ According to the 2011 World Disaster Report, there were more than 700 natural and technological disasters on the average every year in the world between 2001 and 2010, and about 270 million people were affected annually and more than 130 thousand people lost their lives.^2^

The “Critical Success Factors (CSF)” concept was first initiated by Rockart and the Sloan School of Management.CSF are generally used in management areas such as organizational management, operational management and supply chain management.^5^ At the same time, CSF implementation can be used in human resources, information management, internet marketing, tourism sector, new product development, environmental management, public sector and education.^6^ Key success factors can be used instead of each other in terms of CSF in organizational working areas related to management.^7^

Critical success factors (CSF) were introduced in the 1960s as a concept that would help companies achieve their goals and increase their overall competitiveness. In some areas, CSF proactively take the necessary measures to help managers directly influence a particular outcome.^8^

The Federal Emergency Management Agency (FEMA)in 2014 - 2018 Strategic Plan included in its Strategic Prediction part expanding the role of the public in an emergency. Also included in the strategic priorities of the plan was the creation of a “referable organization” to carry out ongoing incident or accident operations appropriately and quickly.^9^

CSF, such as strategic planning, stock management, transportation and capacity planning, information management and technology use, human resource management and continuous improvement and co-operation, obtained from commercial supply chains, are discussed under humanitarian assistance.^8^ The understanding of CSF will contribute to the resolution of some problems for the success of humanitarian supply chains.^10^ CSF is identified in the emergency aid chain in emergencies also.These; routine disaster awareness and training campaigns, special early warning, priority position planning, official management integrity and participation of the military unit.^7^ **Oloruntoba R et al. and the 2009** forest fires in Australia have shown that even in such wealthy countries, they need to increase emergency response and response capacities in disasters.^11^

Zhou et al. conducted a broad literature review of emergency management and summarized the factors that affect emergency management in a tabular form.^5^ In the literature, there are various studies regarding the critical success factors. These studies mostly cover the following areas: commercial, information technologies, total quality management and impact on the performance of critical success factors, enterprise systems, supply chain management, education, project management, enterprise resource planning, emergency logistics and emergency/humanitarian supply chain, manufacturing sector, national emergency management model, emergency relief projects, quality management application, information management, self-governing groups, emergency management.

The aim of this study was to evaluate the critical success factors at disaster and emergency management and to analyse their impact on performance to guide the managers and decision makers.

## METHODS

This study was a cross-sectional study. It included all staff working in the fire brigade, 112 Emergency, Disaster and Emergency Management Authority and Red Crescent institutions serving in emergency situations in five provinces (Rize, Artvin, Trabzon, Gümüşhane, Bayburt) in the Eastern Black Sea region of Turkey. One province (Gümüşhane) was selected by cluster sampling method. In order to be able to carry out our study, necessary permissions were obtained from Gümüşhane Provincial Health Directorate and ethics committee approval was taken. Questionnaire which is the data collection method in the study was applied by face-to-face interview technique by the researchers between 08/01/2016 - 02/02/2016 to all of the staff working in the fire brigade, 112 Emergency, Disaster and Emergency Management Authority and Red Crescent institutions serving in emergency situations in Gümüşhane province. The assessments were made on 92 valid questionnaires. The cronbach’s alfa value of the questionnaire was found to be 0.948. The validity of the questionnaire was determined according to the demographic information of the participants (age, gender, experience etc.) and the completeness of the answers given to the questionnaire. In the survey, CSF in disaster and emergency management is presented under three main headings as planning, response and improvement. 5-point scoring system was used in the study. Likert scale of Five was used in the questionnaire and the meaning of the numbers related to the answer was as the following: 1: Absolutely Disagree, 2: Disagree, 3: Undecided, 4: Agree and 5: Absolutely Agree. CSF in the questionnaire were obtained by translating twenty CSF into Turkish which were used in the study named “Identifying CSFin emergency management using a fuzzy (logic) DEMATEL method” conducted by Zhou, Huang and Zhang.^8^ The evaluation of data was made by statistical package program IBM SPSS Version 22. Factor analysis and Pearson correlation analysis was performed for the statistical analysis.

p ≤0.05 was considered statistically significant. Correlation coefficient is between -1 and +1 (-1≤p≤ + 1). Correlation coefficients between 0.00 and 0.25 expresses ‘very weak’, between 0.26 and 0.49 ‘weak’, between 0.50 and 0.69 ‘medium’, between 0.70 and 0.89 ‘high’, between 0.90 and 1.00 ‘very high’. A positive correlation coefficient indicates that there is a linear relationship between variables and a negative correlation indicates that there is an inverse relationship.

## RESULTS

In the study, 45.7% of the participants were women, 54.3% were men. Again in the study, 14.1% of the survey participants were between the ages of 18-20, 41.3% 21-23, 10.9% 24-26, 8.7% 27-30 and 25% 31+ years. Also 17.4% percent of the respondents had 1-year work experience, 25% two years, 15.2% three years, 7.6% four years and 34.8% had over five years work experience. The results related to the cronbach’s alpha value and planning criterion of the questionnaire are presented in Table-I.

**Table I:** Evaluation of Disaster and Emergency Management Critical Success Planning and Intervention Criteria.

FACTOR (Planning)	X̄	±SD
Well-planned emergency relief supply system	4.55	0.70
Reasonable organizational structure and clear awareness of Responsibilities	4.39	0.73
Applicable emergency response plan and regulations	4.48	0.73
Financial support measures and prior planning of logistic centers and shelters	4.49	0.76
Education campaign on disaster prevention and response	4.46	0.72
Specific training of professionals such as rescue workers and medical Staff	4.51	0.69
Strong ability to send out specific early warning about potential Hazards	4.29	0.83
Regular organization of simulated disaster exercise	4.29	0.73
GENERAL MEAN	4.43	0.74
FACTOR (Response)		±SD
Short-term implementation of emergency plan	4.32	0.80
Government institutions to lead the plan	3.95	0.98
Support and participation of the army	4.02	0.85
Timely and accurate identification of needs	4.49	0.69
The security of aids during distribution and transport	4.49	0.70
Clear procedures of aid delivery and reporting	4.18	0.66
Having an effective emergency information system	4.32	0.69
Implementation of modern logistics technologies	4.33	0.66
GENERAL MEAN	4.26	0.75
FACTOR (Recovery)		±SD
Reconstruction and staff comforting	4.18	0.68
Regular evaluation of statistics and feedback of information of losses and damages	4.25	0.69
Evaluation of the effectiveness and usefulness of the management system	4.25	0.72
Continuous improvement of emergency management operational system	4.39	0.69
GENERAL MEAN	4.27	0.70

According to Table-II, participants rated the planning and response criteria to be the most important for the performance criteria and that the rate of citizens’ preparedness for disasters and emergency situations and the satisfaction rate of emergency or disaster response personnel would increase.

**Table II:** Results Related to the Planning and Response Criteria in the Performance Measures of Disaster and Emergency Management of People in the Research Group.

CRITERIA (Planning)	X̄	±SD
Citizens awareness of disasters and emergenciesincrease	4.35	0.65
Citizens disaster and emergency preparedness rate increases	4.36	0.64
The institutions and organizationsawareness rate of disaster and emergency situations increase	4.33	0.61
The institution and organizations disaster and emergency preparedness rate increases	4.32	0.66
TOTAL	4.34	0.64

*CRITERIA (Response)*	X̄	*±SD*

The citizens satisfaction rate of response to post emergency and disaster increases	4.05	0.63
The citizens satisfaction rate of recovery to post emergency and disaster increases	4.02	0.65
Institutions working in emergency and disaster response increased the rate of positive news in the media	4.01	0.70
Emergency and disaster response staff’s satisfaction rate increases	4.36	0.62
TOTAL	4.11	0.65

There is a significant and positive relationship between all of the critical success factors, planning, response and recovery criteria, and performance variance as shown in Table-III, .It is also evident that the improvement criteria for CSF affects performance.

**Table III:** The Relationship (Correlation) between Critical Success Factors and Performance and the Effect of Critical Success Factors on Performance.

Factor	Planning	Response	Recovery	Performance
Planning		0.76**	0.72**	0.54**
Response	0.76**		0.76**	0.54**
Recovery	0.72**	0.76**		0.61**
Performance	0.54**	0.54**	0.61**	

** p< 0.01.				

*Regression*				

0.399Adjusted, 0.38, F=19.51 p=0.0001				

*Variables*		*Beta Coefficient (ß)*	*tValue*	*p*
Planning		0.167	1.246	0.216
Response		0.093	0.649	0.518
Recovery		0.422	3.124	0.002*

* p< 0.05.

## DISCUSSION

CSF are the key factors that must be applied for success of all organizations. It has been found out that there is a significant and positive relationship between all of the critical success factors, planning, response and recovery criteria, and performance variance.

Although there have been many studies on Critical Success Factors when we look at the studies conducted in the last decade they are as the following; Zhou et al. (2011) CSF in emergency management,^5^ Cochrane (2012) CSF in transforming pedagogy with mobile learning^12^, Chen (2012) CSF in emergency management engineering^13^, CSF in Enterprise Resource Planning applications at small and medium business, Anyanful and Nartey (2015) CSF in the banking sector^14^, Liu et al. (2016) CSF in infrastructure recovery after disaster^15^, Vaz et al. (2016) CSF in emergency management information system^16^, Zhou et al. (2017) CSF in emergency management^17^, Yang et al. (2017) CSF in supply chain management^18^, Nguyen (2017) CSF in logistics and supply chain management^19^, Sheikhi et al. (2017) CSF in the performance of the drug supply chain^20^, Hill (2017) CSF to reduce disasters^21^, Kocak and Diyadin (2018) evaluation of CSF in the transition process of industry to 4.0^22^, Alhamali (2019) CSF in the green supply chain.^23^

In their study, Lu et al. attempted to identify and define CSFin emergency aid logistics.^10^ In this research they focusedon Taiwanese agencies. They have identified 11 CSF such as strategic planning, stock management, transportation and capacity planning, information and human resources management, continuous improvement and cooperation and technology use.^10^ Oloruntoba identified and analyzed some of the key success factors in the emergency aid effort and emergency response chain after a major hurricane in North Australia.^7^ Here the key success factors for the hurricane are divided into two groups. First; preparation of the authorities and effective communication; second the guidance unit are grouped in the integration of government agencies and private sector interventions control of government agencies.^7^ In their work, Galasso and colleagues focused on key performance indicators in disaster management control.^24^ Ab Talib and Abdul Hamid briefly presented in a table the information about the 77 studies in the literature about the CSFin the supply chain in their study.^6^ With these studies, 42 CSFrelated to emergency management and humanitarian aid supply chain were obtained.^6^ Yadav and Barva analyzed the CSF of supply chain in humanitarian aid logistics.^25^ In this study, the direction and sequence of the relationships among these factors were evaluated by Interpretive Structural Modelling method using 12 CSFrelated to supply chains. As a result, it has been found that the “risk and need-setting” factor helps in the success of the “continuous development of preparedness and response practices”. It has also been found that the “sound information and communication technology” factor can be realized with the “official policy and organizational structure” factor.^25^ Using the D-DEMATEL method, Zhou et al. Identified 6 of the most important 10 CSF for emergency management.^17^ All these studies have similar results to our study.

## CONCLUSIONS

As a result of the study, it is seen that the most important CSFs are the factors involved in the planning stage in the institutions and organizations involved in disasters and emergencies. Accordingly, the existence of an effective and successful planning phase in disasters and emergencies will pave the way for success for the institutions and organizations involved in this field. Among the factors in the planning stage, it is seen that the “well-planed emergency relief supply system” factor comes to the forefront more than the other factors, and it is seen that the deficiencies in this area should be solved by the institutions.

On the other hand, among the factors in the intervention phase “Timely and accurate identification of needs” and “The security of aids during distribution and transport” factors are considered to be more important. Among the factors in the recovery phase, the factor “Continuous improvement of emergency management operational system” is more prominent.

It is also noteworthy that all three of the main factors in the planning and response stages of disaster and emergency management are related to emergency logistics. This situation shows the importance of logistics activities in disaster and emergency management. Therefore, while giving more importance to this field, disasters and emergencies will be managed more successfully and effectively, taking into consideration the weight of other CSFs.

### Authors’ Contribution:

**SS** conceived, designed and did statistical analysis & editing of manuscript, is responsible for integrity of research.

**UY & TS** did data collection and manuscript writing.

**SS** did review and final approval of manuscript.
